# Supplementary material for: Assessing the Importance of Native Mycorrhizal Fungi to Improve Tree Establishment after Wildfires
Source: J Fungi (Basel). 2023 Mar 29;9(4):421. doi: 10.3390/jof9040421 (PMC10144394; doi:10.3390/jof9040421)

Table S1. a) Frequency of occurrence (%) of mycorrhizal structures and fungal endophytes present in four trees of the Chilean matorral in both inoculated (M+) and non-inoculated (M-) treatment. b) Results of Randomization test (Rand. Test) comparing the frequency of occurrence of mycorrhizal structures and fungal endophytes present in four trees of the Chilean matorral. For each tree species were selected five individuals and five different roots from each of these individuals (total n = 25 samples).

**a)**	**M+**		**M-**	
**Species**	**Mycorrhizal**	**Endophytes**	**Mycorrhizal**	**Endophytes**
Cryptocaria alba	88 ±10.9	24 ±8.9	4 ±8.9	4 ±10.9
Kageneckia oblonga	84 ±8.9	20 ±19.7	4 ±8.9	8 ±11.0
Peumus boldus	88 ±10.9	44 ±16.7	0 ±0.0	12 ±10.9
Quillaja saponaria	92 ±10.9	24 ±8.9	4 ±10.9	4 ±10.9

**b)**	**Mycorrhizal**			**Endophytes**		
**Species**	**M+**	**M-**	**Rand. test**	**M+**	**M-**	**Rand. test**
Cryptocaria alba	22	1	**$p < 0.0001$**	6	1	$p = 0.095$
Kageneckia oblonga	21	1	**$p < 0.0001$**	5	2	$p = 0.398$
Peumus boldus	22	0	**$p < 0.0001$**	11	3	**$p = 0.041$**
Quillaja saponaria	23	1	**$p < 0.0001$**	6	1	$p = 0.095$

Supplementary Figure S1. Enzymatic activity for dehydrogenase (D), β-glucosidase (G) and urease (U) in four dominant woody plant species from Chilean Mediterranean Ecosystem (MTE) after a fire, measured after 24 months. M+ = plant inoculated with arbuscular mycorrhizal fungi. M- = uninoculated plants. Significant differences (pairwise t-test; Bonferroni correction) between treatments are represented with $^*p<0.05$; $^{**}p<0.01$; $^{***}p<0.001$

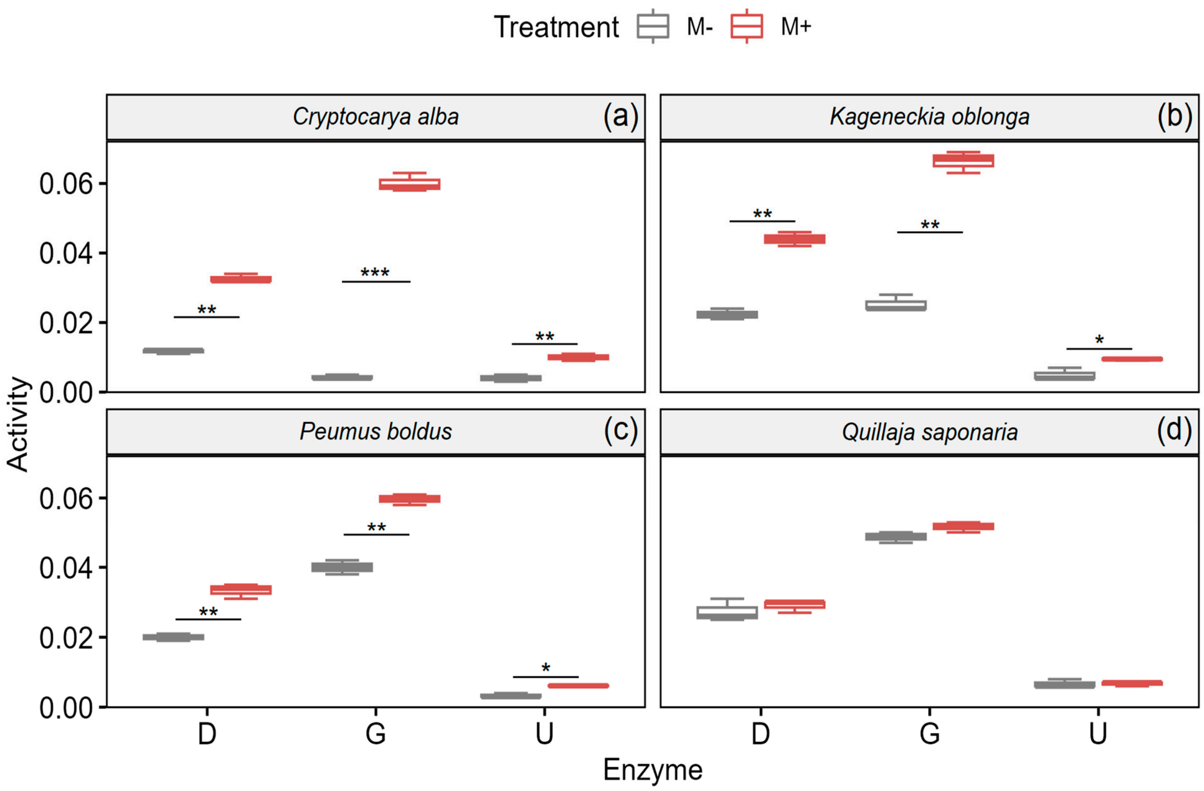

Supplementary Figure S2. Soil macronutrients (nitrogen, phosphorus and potassium) in four dominant woody plant species from Chilean Mediterranean Ecosystem (MTE) after a fire, measured after 24 months. M+ = plant inoculated with arbuscular mycorrhizal fungi. M- = uninoculated plants. Significant differences (pairwise t-test; Bonferroni correction) between treatments are represented with *p<0.05; **p<0.01; ***p<0.001

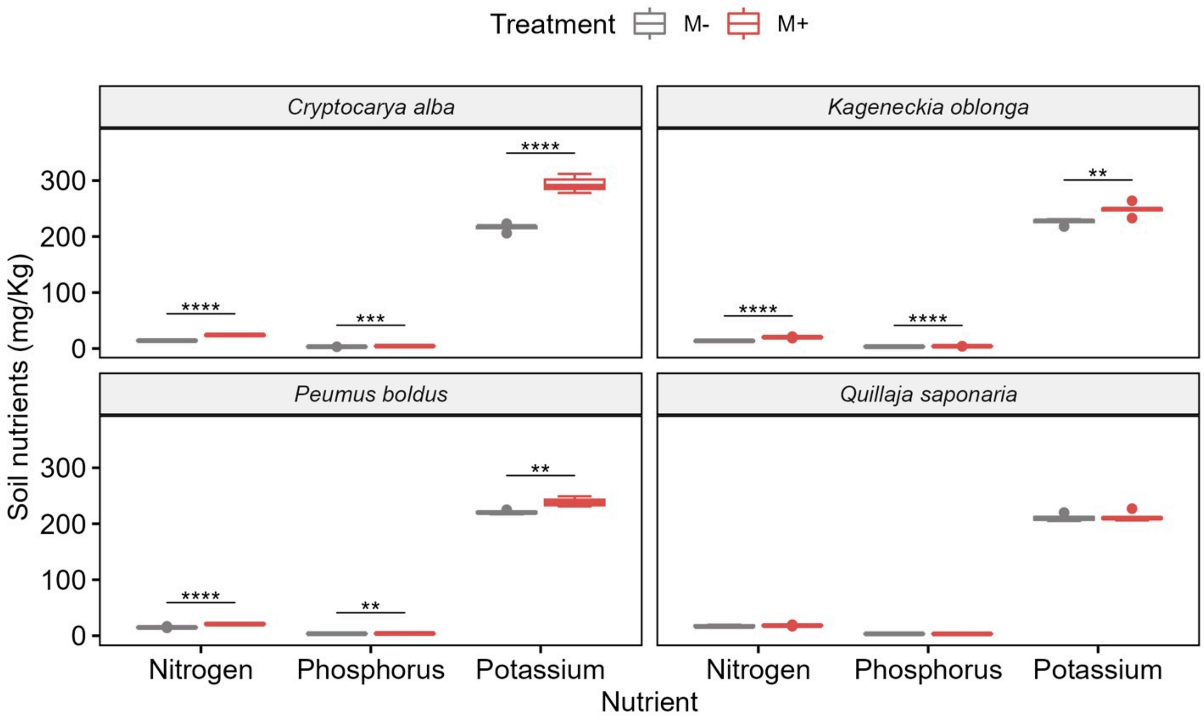

Supplement: Supplementary file 1 [file jof-09-00421-s001.zip › jof-2243585-supplementary.pdf]
